# Efficacy of diet restriction with or without probiotic for treatment of patients with IBS‐D: Phase I−II clinical trial

**DOI:** 10.1002/iid3.857

**Published:** 2023-05-08

**Authors:** Xian‐Shu Zhao, Li‐Jun Shi, Bao‐Li Ning, Zhi‐Ming Zhao, Xiao‐Xue Li, Meng‐Hua Zhu, Ya‐Bing Zhang, Jun Fu

**Affiliations:** ^1^ Health Center of Screening and Prevention of Diseases First Affiliated Hospital of Harbin Medical University Harbin China; ^2^ Department of gastroenterology First Affiliated Hospital of Harbin Medical University Harbin China

**Keywords:** Bifidobacteria, diarrhea‐predominant irritable bowel syndrome, IBS‐SSS, IgG antibody titers, probiotic, restricted diet

## Abstract

**Background and Aim:**

Diet is a major contributor to irritable bowel syndrome (IBS) and is also a powerful tool for treatment of IBS. This study compared two diets and explored the effectiveness of the diets when combined with a probiotic for treatment of IBS‐D patients.

**Methods:**

Phase I, patients were randomized into groups; control, cold/spicy/fried restricted diet (CSF res diet), IgG positive restricted diet (IgG res diet), and a combination both diets (CSF + IgG res diet). Phase II, patients were randomized into IgG res diet + placebo and IgG res diet + probiotic. Both interventions were 12 weeks in duration. Symptom Severity Scale (IBS‐D‐SSS) and IgG titer were assessed at the beginning and the end of the study.

**Results:**

Totals of 214 and 167 patients completed the two parts of the study, respectively. After intervention, IBS‐D‐SSS and TIgG grade were significantly improved compared to baseline, with results similar to the control group. In general, there were decreases in IBS‐D‐SSS and TIgG grade that were significantly different among the groups. There were exceptions; no differences were observed for IBS‐D‐SSS between the IgG res diet and CSF + IgG res diet, or TIgG grade between the CSF res diet, IgG res diet, and CSF + IgG res diet. However, the CSF res diet and IgG res diet had a synergistic effect that decreased IBS‐D‐SSS and TIgG titer, with a greater contribution by the IgG res diet. Therefore, we evaluated the IgG res diet with either placebo or probiotic and found that IBS‐D‐SSS and TIgG grade decreased from baseline. There was a significant decrease in IBS‐D‐SSS with the probiotic but TIgG grade was not significantly different between the IgG diet + placebo and IgG diet + probiotic diet.

**Conclusions:**

Both the CSF res diet and IgG res diet improved IBS symptoms and demonstrated synergy, although the IgG res diet had a greater contribution. Further, when intolerant foods cannot be eliminated from a diet, avoiding uncooked, cold, spicy, fried, and alcoholic foods is a superior choice. The IgG res diet combined with Bifidobacteria was the best dietary choice and may function though a non‐IgG pathway.

## Graphical abstract

We explored a way to accurately choose a restricted diet and then combined with probiotics, so that the discomfort symptoms of irritable bowel syndrome can be far away from patients, and at the same time, it will not lead to malnutrition or intestinal flora imbalance due to excessive fasting.

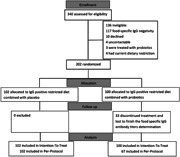

## 1 INTRODUCTION

Irritable bowel syndrome (IBS) is a functional intestinal disorder that affects 5%–20% of the general population.^1^, ^2^, ^3^, ^4^, ^5^ This condition reduces considerably a patients' quality of life. IBS does not typically progress to serious illness or death.^1^, ^6^, ^7^ The etiopathogenesis and pathophysiology of IBS are not clear and likely include many different factors such as visceral pain hypersensitivity, improper immune activation, colon dysmotility, psychological conditions, and a history of gastrointestinal infections.^8^, ^9^ IBS patients suffer from intermittent abdominal pain/discomfort, altered bowel habits, and abdominal bloating/distension.^10^ Patients believe that their symptoms are triggered by certain food items such as milk and milk products, wheat products, caffeine, cabbage, onion, peas, beans, hot spices, fried, and smoked food.^11^ It is noteworthy that 20%–70% of IBS patients complain of subjective intolerance to various foods.^12^ As such, diet likely plays a major role in the pathophysiology of IBS^13^, ^14^ and may be a means by which to manage IBS.^15^ Dietary management of IBS has not been carefully assessed, although various food exclusion strategies such as gluten restriction or lactose avoidance have been evaluated. However, convincing evidence that such exclusion strategies are clinically useful is lacking.^16^, ^17^ and may only have a partial effect. Further, excessive food restriction can result in an imbalance of the intestinal flora as well as malnutrition. One study found that 62% of IBS patients had either limited or excluded certain food items from their daily diet and of those, 12% were at risk for long‐term nutritional deficiencies.^18^ Therefore, an effective solution to management of IBS should include an accurate selection of a restricted diet and the appropriate implementation of effective probiotics.

According to the International Scientific Association for Probiotics and Prebiotics (ISAPP), probiotics are defined as “live microorganisms that, when administered in adequate amounts, confer a health benefit on the host”.^19^ Probiotics have been used clinically to improve the symptoms of IBS patients for some time,^20^, ^21^, ^22^, ^23^ although the precise efficacy of probiotics for IBS is largely unknown. For the management of IBS, it is unclear whether particular combinations of probiotics are more effective than others, or whether there are particular IBS subtypes that are more responsive to probiotics. Individual probiotic strains may differ in their effectiveness. Therefore, it is essential to assess the effectiveness of individual strains, in that pooling of results from studies examining a particular genus and species may mask the beneficial effect of individual probiotic strains.^10^ Many probiotic preparations used to manage IBS symptoms contain Bifidobacteria,^24^, ^25^ which suggests the potential usefulness of Bifidobacteria for the treatment of IBS patients.

The aim of this study was to assess the effects of dietary restriction and probiotic use on IBS‐D patients. Assessment was based on positive food IgG antibody testing and cold/spicy/fried dietary restriction during a 12‐week intervention. Results of this study provide insight into a more accurate and effective approach for IBS patient treatment and management.

## 2 MATERIALS AND METHODS

### 2.1 Study design and patients

We performed a 2 × 2 factorial design, single‐center, randomized trial with a 12‐week dietary intervention for the first part of the study (2013–2015) and proceeded to conduct a randomized, double‐blind, placebo‐controlled trial for the second part of the study. The study consisted of a 12‐week restricted diet combined with placebo or probiotics for patients (recruited from 2016 to 2019) with an IBS‐D diagnosis. Patients were recruited from the gastroenterology clinic of the First Affiliated Hospital of Harbin Medical University. Although Rome IV was published in 2016, the diagnostic criteria of Rome III were applied to both parts of the study to maintain consistency. Inclusion criteria: (1) female or male 18–65 years of age; (2) IBS‐D diagnosis by the Rome III criteria; and (3) positivity for food‐specific serum antibody. Exclusion criteria: (1) unable to follow the research plan; (2) report of an IgE‐mediated food allergy; (3) continued use of other medications, probiotics or ongoing restrictive exclusion diet; and (4) major medical conditions such as gastrointestinal surgery, diabetes, or inflammatory bowel disease.

Clinical characteristics (age, gender, body mass index, smoking, and symptom duration), 3‐day food diary (2 working days and 1 holiday), 7‐day Symptom Severity Scale (IBS‐D‐SSS) (Table 1), and food specific IgG antibody titers were obtained at baseline. Personalized food elimination plans were given to patients. Patients were contacted by telephone once a week during the 12‐week intervention for collection of diet diary entries, foods eaten, and compliance. At the end of the study, 7‐day IBS‐D‐SSS and IgG titers were assessed. Adverse events were recorded at weekly telephone calls and at the follow‐up visit.

**Table 1:** IBS‐D symptom severity scale (IBS‐D‐SSS).

Symptoms	Severity	Scale
Stool consistency	Smooth and soft	0
	In soft clumps with clear edges	1
	Pasty	2
	Watery	3
Abdominal pain/distension	Without symptoms during the week	0
	With symptoms but without impact on normal life and work	1
	With symptoms and impact on normal life and work, but not significant	2
	With great impact on normal life and work	3
Defecation frequency	0–2 times/day	0
	3–4 times/day	1
	5–7 times/day	2
	More than 7 times/day	3

### 2.2 Intervention

For the first part of the study, participants were randomly divided into four groups: no dietary restriction (control), cold/spicy/fried restricted diet (CSF res diet), IgG positive food restricted diet (IgG res diet), and both restricted diets (CSF + IgG res diet). The interventions were as follows: control patients were requested to follow an unchanged normal (China/Eastern) diet (Table 2) during the 12‐week study. CSF res diet: the IgG positive food was not restricted but cold/spicy/fried food was restricted. IgG res diet: the IgG positive food was restricted whereas the CSF diet was not. CSF + IgG res diet: the IgG positive food and the CSF diet were both restricted. The diet intervention lasted for 12 weeks. A restricted diet may influence nutritional status, therefore a dietitian evaluated and controlled the patient's diet as described in (Table 2).

**Table 2:** Specified food list.

Unchanged normal (China/Eastern) diet containing in no CSF res diet groups		
Raw and cold food	Cold food and drinks	
	Raw vegetable/meat/poultry/seafood/fish	
	Ice cream	
Spicy food	Spicy spices: chilies, mustard, Chinese prickly ash, pepper, curries, horseradish, and so forth.	
	Spicy vegetables: Leek, pepper, onion, scallion, garlic, ginger, and so forth.	
	Liquor, wine, rice wine, Shaoxing wine, wine, and so forth.	
Fried food	Fried fruits，Fried vegetables，fried dough twist, fried spring rolls, fried balls, fried dough sticks, fried chicken, fried steak, fried pork chops, fried fish, and so forth.	
Sham diet‐alternative food in IgG res diet group		
Allerquant™ 14 Foods IgG Elisa Kit Food		Alternative Food
Cow's Milk		Goat's milk
Wheat		Oats
Beef		Mutton
Chicken		Duck
Codfish		Hairtail
Corn		Oats
Crab		Scallops
Eggs		Cheese
Mushroom		Kelp
Pork		Turkey
Rice		Potatoes or Millet or Oats
Shrimp		Clam
Soybean		Oats
Tomato		Carrots

For the second part of the study, patients were randomly divided into two groups: IgG res diet + placebo and IgG res diet+probiotic. Each study capsule contained 0.5 billion *Bifidobacterium adolescentis* and each participant ingested either *Bifidobacterium* capsules or four identical placebo capsules, twice daily, for 12 weeks. Patients avoided any hot beverage (tea/coffee) for at least 30 min after ingestion of probiotic or placebo.

### 2.3 Randomization and masking

For the first part of the study, patients were randomized to one of the four study groups based on computer generated random numbers in a 1:1:1:1 ratio using a random block size of four. For the second part of the study, patients were randomized to one of the two groups based on computer generated random numbers in a 1:1 ratio using a random block size of two. The random number list was generated by QuickCalc GraphPad Software Inc. The randomization was performed by an independent statistician. Allocation concealment was done by sequentially numbered sealed opaque envelopes. The diet allocation was concealed in an opaque envelope that was only opened after all baseline data had been collected. The randomization was blinded for both the patients and the investigators. Allocation to diet was masked throughout data collection, laboratory analysis, data input, and data analysis.

### 2.4 Measurements

The primary outcomes were a decrease in IBS‐D‐SSS and TIgG antibody titer at completion of the study compared to baseline. The secondary outcome was percent decrease in IgG antibody titer.

### 2.5 IBS‐D symptom severity scale

IBS‐D‐SSS (Table 1) was developed to assess patient symptoms for 7 consecutive days before baseline and at the end of the study. The IBS‐D‐SSS includes three items (stool consistency, abdominal pain, and defecation frequency distension) with total scores ranging from 0 to 9 and each item scored on a scale from 0 to 3. Reduction in IBS‐D‐SSS after intervention was calculated and compared.

### 2.6 TIgG antibody titer determination

Four milliliters of venous blood from each subject was drawn in the early morning at baseline and at the end of the study. Serum was used for testing. IgG antibodies reactive with 14 types of food (cow's milk, wheat, beef, chicken, codfish, corn; crab, eggs, mushroom, milk, pork, rice, shrimp, soybean, and tomato) were semi‐quantitatively detected with the IgG food testing kit produced by Biomerica (Allerquant™ 14 Foods IgG Elisa Kit). An IgG antibody titer of greater than 50 U/mL for one of the foods was defined as a positive IgG titer to that food. For each patient tested, the positive IgG antibody titers of all 14 foods tested with the kit were added together. The resulting value was designated as ‘total positive IgG antibody titer (TIgG titer)’ for that patient. The percent decrease in TIgG titer was calculated by dividing the difference between the original and final TIgG titer by the original titer and multiplication by 100%. TIgG titer < 50 U/mL, 50 U/mL ≤ TIgG titer < 100 U/mL, 100 U/mL ≤ TIgG titer < 200 U/mL and TIgG titer ≥ 200 U/mL were designated as TIgG titer grades: negative, mild, moderate, and severe, respectively.

### 2.7 Statistical analysis

Both Intention‐To‐Treat and Per‐Protocol analyses were considered. Continuous data were expressed as means and standard deviation, with categorical variables expressed as number and percentage. Analysis of variance, Chi‐square, Fisher's exact, and Kruskal‐Wallis tests were used to compare baseline factors among groups. Bonferroni, Chi‐square, and Fisher's exact tests were used for pairwise comparisons. A paired t test and Wilcoxon's sign rank test were used for comparison of the same group before and after treatment. Kruskal‐Wallis, Fisher's exact, and Chi‐square tests were used to compare end points among each group. Collected data were analyzed using SPSS version 24 (SPSS Inc.). Statistical significance was defined as a two‐tailed *p* < .05.

### 2.8 Ethics approval and consent to participate

The ethics and protocol of this research study were assessed and approved by the Ethics Committee of the First Affiliated Hospital of Harbin Medical University (No.201324). This study was conducted in accordance with the Declaration of Helsinki. We discussed the research protocol and the potential risks with each patient. All patients provided written informed consent. This study has been registered in the Chinese Clinical Trial Registry (Registration number: ChiCTR2000029107).

## 3 RESULTS

### 3.1 Patients

**Figure 1:**
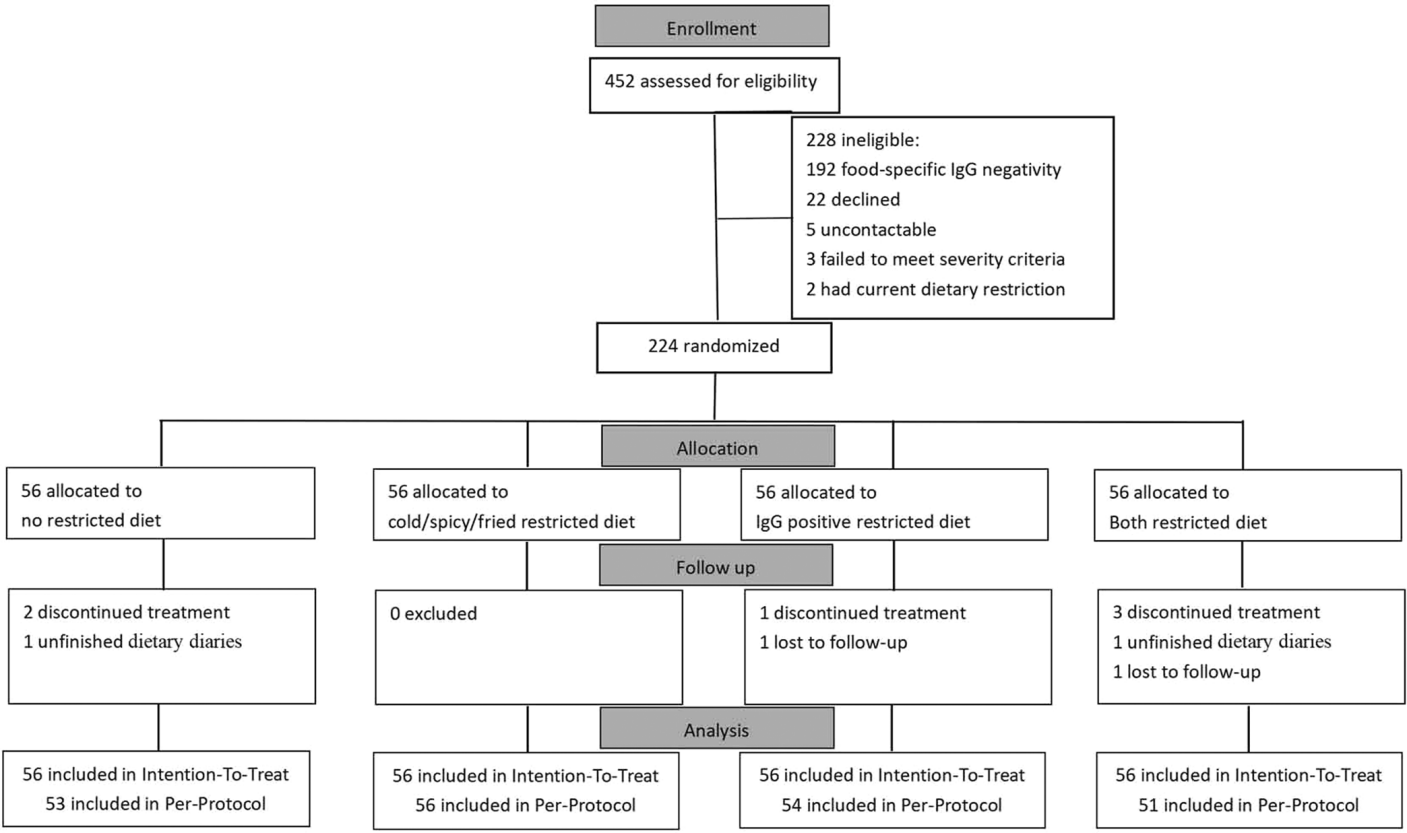
Flow diagram of 2 × 2 factorial design and randomized trial with two restricted diets.

Of 452 IBS‐D patients assessed for eligibility, 224 patients were randomized to four groups as follows: control (*n* = 56), CSF res diet (*n* = 56), IgG res diet (*n* = 56), and CSF + IgG res diet (*n* = 56). Of these, 192 patients were ineligible due to a lack of food‐specific IgG, 22 for refusing to participate in or coordinate with the study, five could not be contacted, three for failure to meet severity criteria, with two excluded because of current dietary restrictions. All 224 randomized patients were included in the Intention‐To‐Treat analysis and 214 were included in the Per‐Protocol analysis. Ten patients were excluded from the Per‐Protocol analysis: six subjects completed less than 80% of the intervention (two control, one IgG res diet, three CSF + IgG res diet), two subjects failed to complete dietary diaries (one control, one IgG res diet), and two subjects failed to accept return visits (one IgG res diet, one CSF + IgG res diet). (Figure 1)

**Figure 2:**
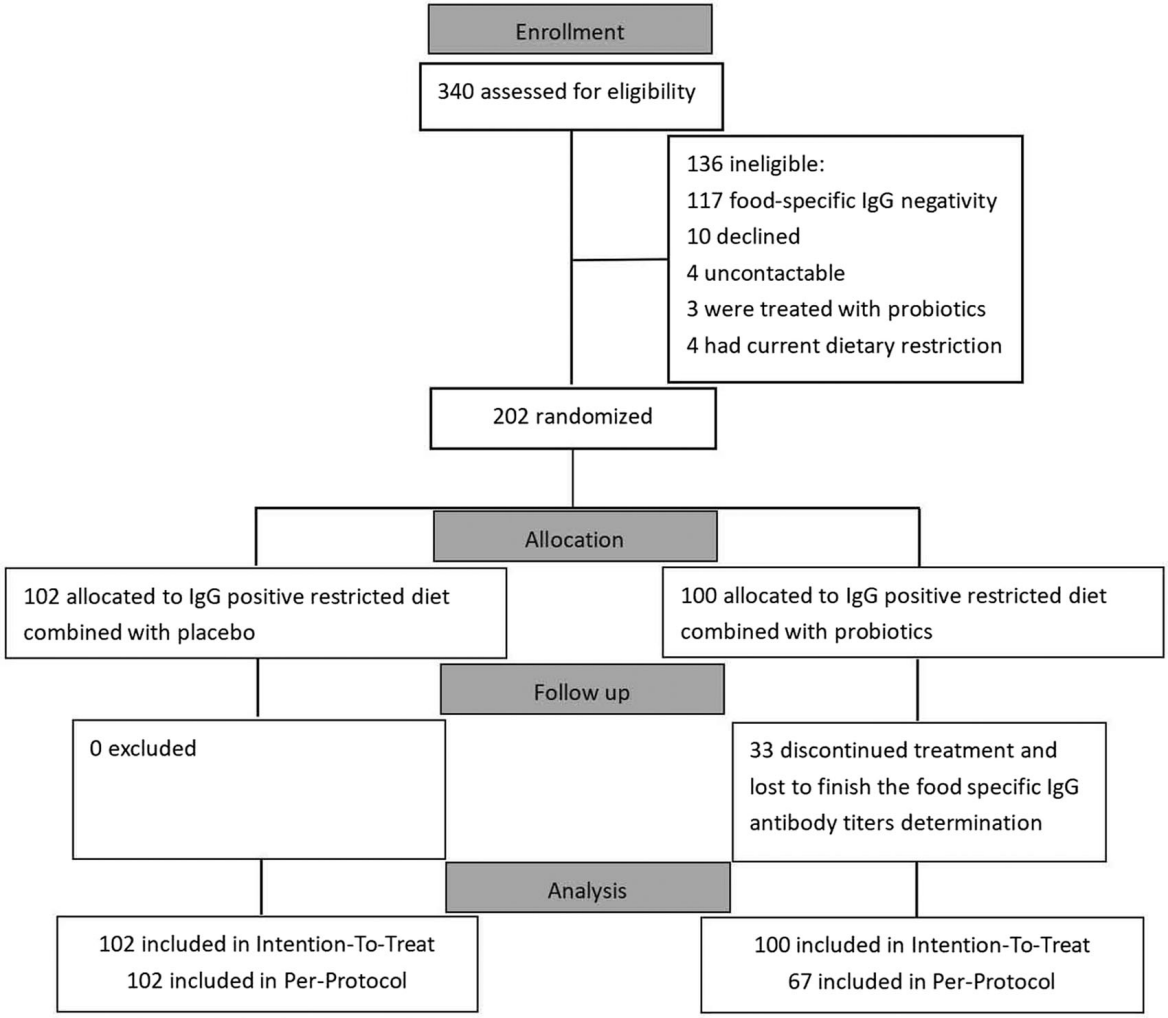
Flow diagram of IgG positive restricted diet combined with placebo or probiotics.

We recruited another 340 IBS‐D patients. After determination of eligibility, 202 patients were randomized to IgG positive food dietary restriction combined with probiotic or placebo: IgG res diet + placebo (*n* = 100) vs IgG res diet+probiotic (*n* = 102). Of these, 117 patients were ineligible due to a lack of food‐specific IgG, 10 for refusing to participate in or coordinate with the study, four could not be contacted, three for current treatment with probiotic, and four for a current diet restriction. All 202 randomized patients were included in the Intention‐To‐Treat analysis and 169 were included in the Per‐Protocol analysis. Of these 33 patients were excluded from the Per‐Protocol analysis for declining to finish the food specific IgG antibody titer determination. (Figure 2)

There were no significant differences among the first four groups or between the latter two groups with regard to; demographic data, anthropometric measurements, clinical characteristics, IBS‐D‐SSS at baseline, and TIgG titer at baseline. (Table 3).

**Table 3:** Patient characteristics at baseline.

2 × 2 factorial design, randomized trial with two restricted diet						IgG restricted diet combined with placebo or probiotics		
	Control	CSF diet	IgG diet	CSF + IgG diet	*p* Value	IgG diet + placebo	IgG diet + probiotics	*p* Value
Intention‐to‐treat								
Number	56	56	56	56		100	102	
Age (y)	37.0 ± 9.5	38.3 ± 6.7	36.7 ± 7.1	35.7 ± 8.0	0.385^a^	34.0 ± 9.2	35.8 ± 8.0	0.130^b^
Gender (%Female)	36 (64.3%)	35 (64.3%)	34 (60.8%)	36 (64.3%)	0.976^c^	61 (61.0%)	62 (60.8%)	0.975^c^
BMI (kg/m^2^)	23.4 ± 3.7	24.4 ± 3.0	23.4 ± 4.0	24.8 ± 4.0	0.100^a^	24.1 ± 3.8	25.0 ± 3.9	0.136^b^
Smoker (yes%)	5 (8.9%)	4 (7%)	4 (7%)	4 (7%)	0.979^c^	7 (7.0%)	8 (7.9%)	0.819^c^
Symptom duration (months)	18 (14–23.8)	20.5 (14–26)	19 (15.3–24.8)	21 (15–25)	0.737^d^	15 (12.3–17)	15 (11.8–19)	0.393^e^
IBS‐D‐SSS	5.3 ± 0.1	5.4 ± 0.5	5.5 ± 0.3	5.4 ± 0.4	0.172^a^	5.4 ± 0.5	5.3 ± 0.2	0.205^b^
TIgG titers grade								
Negative	0	0	0	0	0.925^c^	0	0	0.694^c^
Mild	29	28	27	26		46	51	
Moderate	15	11	14	13		20	22	
Severe	12	17	15	17		34	29	
Per‐Protocol								
Number	53	56	54	51		67	102	
Age (y)	37.2 ± 9.7	38.3 ± 6.7	36.6 ± 7.2	35.5 ± 8.3	0.447^a^	34.7 ± 8.9	35.8 ± 8.0	0.407^b^
Gender (%Female)	34 (64.2%)	35 (62.5%)	32 (59.3%)	33 (64.7%)	0.938^c^	40 (59.7%)	62 (60.8%)	0.888^c^
BMI (kg/m2)	23.4 ± 3.7	24.4 ± 3.0	23.5 ± 4.0	25.0 ± 4.0	0.094^a^	23.9 ± 3.5	25.0 ± 3.9	0.076^b^
Smoker (yes%)	4 (7.5%)	4 (7.1%)	4 (7.4%)	3 (5.9%)	0.987^c^	5 (7.5%)	8 (7.9%)	0.928^c^
Symptom duration (months)	18 (14–24.5)	20.5 (14–26)	19 (15.8–25)	21 (15–25)	0.840^d^	15 (13–18)	15 (11.8–19)	0.760^e^
IBS‐D‐SSS	5.3 ± 0.1	5.4 ± 0.5	5.5 ± 0.3	5.4 ± 0.5	0.161^a^	5.3 ± 0.5	5.3 ± 0.2	0.744^b^
TIgG titers grade								
Negative	0	0	0	0	0.909^c^	0	0	0.822^c^
Mild	28	28	25	23		32	51	
Moderate	13	11	14	11		13	22	
Severe	12	17	15	17		22	29	

*Note*: Data are mean ± SD, *n* (%) or median (25th, 75th percentile).

Abbreviations: ANOVA, analysis of variance; BMI, body mass index; IBS‐D‐SSS, diarrhea‐predominant irritable bowel syndrome Symptom Severity Scale; IgG diet, the restricted diet based on food IgG antibodies; TIgG titer, total positive IgG antibody titers. CSF diet, cold/spicy/fried restricted diet.

^a^ one‐way ANOVA test.

^b^ independent *t* test.

^c^ Chi square test.

^d^ Kruskal−Wallis test.

^e^ Mann–Whitney *U* test.

### 3.2 2 × 2 factorial design and randomized trial with two restricted diets

#### 3.2.1 IBS‐D symptom severity scale

The results of IBS‐D‐SSS were consistent for both Intention‐To‐Treat and Per‐Protocol analysis. IBS‐D‐SSS reduction was significant among groups (*p* < .001), with the exception of the IgG res diet and CSF + IgG res diet (4.0 ± 0.6 vs. 4.3 ± 0.5, *p* = .882). IBS‐D‐SSS decreased significantly for each group from baseline to the end of the study (*p* < .001), with mean levels similar to the control group (5.3 ± 0.1 vs. 5.2 ± 0.2). The degree of improvement was control < CSF res diet < IgG res diet < CSF + IgG res diet. (Table 4)

**Table 4:** Comparison of patient IBS‐d‐SSS and TIgG titer.

2 × 2 Factorial design, randomized trial with two restricted diet							IgG restricted diet combined with placebo or probiotics		
		Control	CSF diet	IgG diet	CSF + IgG diet	p value	IgG diet + placebo	IgG diet + probiotics	*p* Value
Intention‐To‐Treat									
Number		56	56	56	56		100	102	
IBS‐d‐SSS									
Baseline		5.3 ± 0.1	5.4 ± 0.5	5.5 ± 0.3	5.4 ± 0.4	.172^a^	5.4 ± 0.5	5.3 ± 0.2	.205^b^
Decrease from baseline		0.1 ± 0.2	3.4 ± 0.6	4.0 ± 0.6	4.3 ± 0.5	<.001^a^	3.2 ± 1.3	3.8 ± 0.6	<.001^b^
*p* value		<.001^c^	<.001^c^	<.001^c^	<.001^c^		<.001^c^	<.001^c^	
TIgG titers grade									
Baseline	Negative	0	0	0	0	.925^d^	0	0	.694^d^
	Mild	29	28	27	26		46	51	
	Moderate	15	11	14	13		20	22	
	Severe	12	17	15	17		34	29	
End of study	Negative	3	18	29	23	<.001^d^	34	43	.414^d^
	Mild	24	22	16	21		34	34	
	Moderate	16	12	5	10		19	18	
	Severe	13	4	6	2		13	7	
p value		.315^d^	.001^d^	<.001^d^	<.001^d^		<.001^d^	<.001^d^	
Per‐Protocol									
Number		53	56	54	51		67	102	
IBS‐D‐SSS									
Baseline		5.3 ± 0.1	5.4 ± 0.5	5.5 ± 0.3	5.4 ± 0.5	.161^a^	5.3 ± 0.5	5.3 ± 0.2	.744^b^
Decrease from baseline		0.1 ± 0.2	3.4 ± 0.6	4.0 ± 0.6	4.3 ± 0.5	<0.001^a^	3.1 ± 1.2	3.8 ± 0.6	<.001^b^
*p* value		<.001^c^	<.001^c^	<.001^c^	<.001^c^		<.001^c^	<.001^c^	
TIgG titers grade									
Baseline	Negative	0	0	0	0	.909^d^	0	0	.822^d^
	Mild	28	28	25	23		32	51	
	Moderate	13	11	14	11		13	22	
	Severe	12	17	15	17		22	29	
End of study	Negative	3	18	29	23	<.001^d^	34	43	.351^d^
	Mild	24	22	14	18		20	34	
	Moderate	15	12	5	8		12	18	
	Severe	11	4	6	2		1	7	
*p* Value		.322^d^	<.001^d^	<.001^d^	<.001^d^		<.001^d^	<.001^d^	

*Note*: Data are mean ± SD, *n* or median (25th，75th percentile).

Abbreviations: ANOVA, analysis of variance; CSF diet, cold/spicy/fried restricted diet; IBS‐D‐SSS, diarrhea‐predominant irritable bowel syndrome Symptom Severity Scale; IgG diet, the restricted diet based on food IgG antibodies; TIgG titer, total positive IgG antibody titers.

^a^ One‐way ANOVA test.

^b^ Independent *t* test.

^c^ Dependent *t* test.

^d^ Chi square test.

#### 3.2.2 TIgG titer grade

TIgG titer grade results were consistent for both Intention‐To‐Treat and Per‐Protocol analysis. At the end of the study, TIgG titer grade significantly decreased from baseline (*p* < .05) for each group with the exception of the control group. The TIgG titer grade for each of the other three groups (after intervention) was significantly lower than the control group (*p* < .001). However, there was no significant difference among the three restricted diet groups. (Table 4)

#### 3.2.3 Interaction effect of the CSF res diet and IgG res diet on IBS‐D‐SSS

Both the CSF res diet and IgG res diet contributed significantly to IBS‐D‐SSS reduction (*p* < .001). Reductions in IBS‐D‐SSS were most apparent in patients with the IgG res diet. No significant effect was observed for the IgG res diet combined with the CSF res diet (4.0 ± 0.6 vs. 4.3 ± 0.5, *p* = .882). The IgG res diet was the main effect factor. (Table 5)

**Table 5:** The effects of CSF res diet and/or IgG res diet on IBS‐D‐SSS after intervention.

		IgG res diet			Total
		No (*n* = 107)	Yes (*n* = 107)	*p* yes versus no	
CSF res diet	No (*n* = 109)	0.1 ± 0.2	4.0 ± 0.6^b^	<0.001	2.1 ± 2.0
	Yes (*n* = 105)	3.4 ± 0.6^a^	4.3 ± 0.5	<0.001	3.8 ± 0.7^d^
	*p* yes versus no	<0.001	0.882	*p*_a versus b_ < 0.001	<0.001
Total		1.8 ± 1.7	4.1 ± 0.5^c^	<0.001	*p*_c versus d_ < 0.001
CSF res diet main effect		*p* < .001			
IgG res diet main effect		*p* < .001			
Interaction effect		*p* < .001			

*Note*: Data are mean ± SD. General linear models (Two‐way ANOVA) were used to analyze the main effects and interaction effect of two interventions. The statistics of the Intention‐To‐Treat and Per‐Protocol were the same.

Abbreviations: ANOVA, analysis of variance; SD, standard deviation.

^a^ Decrease of IBS‐D‐SSS in patients with CSF res diet but no IgG res diet.

^b^ Decrease of IBS‐D‐SSS in patients with IgG res diet but no CSF res diet.

^c^ Decrease of IBS‐D‐SSS in patients with IgG res diet regardless of CSF res diet.

^d^ Decrease of IBS‐D‐SSS in patients with CSF res diet regardless of IgG res diet.

#### 3.2.4 Interaction effect of the CSF res diet and IgG res diet on TIgG titer

Both the CSF res diet and IgG res diet contributed significantly to the percent decrease in TIgG titer (*p* < .001). Reductions in TIgG titer were most apparent for patients with the IgG res diet. The IgG res diet was the main effect factor. (Table 6)

**Table 6:** The effects of CSF res diet and/or IgG res diet on TIgG titer after intervention.

Intention‐to‐treat		IgG res diet			Total
		No (n = 112)	Yes (n = 112)	*p* yes versus no	
CSF res diet	No (*n* = 112)	10.7 ± 17.1	57.3 ± 21.3^b^	<0.001	34.0 ± 30.3
	Yes (*n* = 112)	50.2 ± 19.7^a^	55.5 ± 26.2	1.000	52.8 ± 23.2^d^
	*p* yes versus no	<0.001	1.000	***p***_a versus b_ **=** 0.781	<0.001
Total		30.5 ± 27.0	56.4 ± 23.8^c^	<0.001	***p***_c versus d_ = 0.257
CSF res diet main effect		*p* < .001			
IgG res diet main effect		*p* < .001			
Interaction effect		*p* < .001			

Per‐Protocol		IgG res diet			Total
		No (*n* = 107)	Yes (*n* = 107)	*p* yes versus no	
CSF res diet	No (*n* = 109)	11.3 ± 17.4	59.5 ± 18.5 f	<0.001	35.6 ± 30.1
	Yes (*n* = 105)	50.2 ± 19.7^e^	60.9 ± 20.4	0.160	55.3 ± 20.6 h
	*p* yes versus no	<0.001	1.000	***p***_e versus f_ = 0.316	<0.001
Total		31.3 ± 26.9	60.2 ± 19.4 g	<0.001	***p***_g versus h_ = 0.078
CSF res diet main effect		*p* < .001			
IgG res diet		*p* < .001			
Interaction effect		*p* < .001			

*Note*: Data are mean ± SD. General linear models (Two‐way ANOVA) were used to analyze the main effects and interaction effect of two interventions.

Abbreviations: ANOVA, analysis of variance; SD, standardard deviation.

^a^ % decrease of TIgG titres in patients with CSF res diet but no IgG res diet in Intention‐To‐Treat.

^b^ % decrease of TIgG titres in patients with IgG res diet but no CSF res diet in Intention‐To‐Treat.

^c^ % decrease of TIgG titres in patients with IgG res diet regardless of CSF res diet in Intention‐To‐Treat.

^d^ % decrease of TIgG titres in patients with CSF res diet regardless of IgG res diet in Intention‐To‐Treat.

^e^ % decrease of TIgG titres in patients with CSF res diet but no IgG res diet in Per‐Protocol.

^f^ % decrease of TIgG titres in patients with IgG res diet but no CSF res diet in Per‐Protocol.

^g^ % decrease of TIgG titres in patients with IgG res diet regardless of CSF res diet in Per‐Protocol.

^h^ % decrease of TIgG titres in patients with CSF res diet regardless of IgG res diet in Per‐Protocol.

## 4 IGG POSITIVE RESTRICTED DIET COMBINED WITH PLACEBO OR PROBIOTIC

### 4.1 IBS‐D symptom severity scale

Statistical results for the Intention‐To‐Treat and Per‐Protocol analysis were similar. IBS‐D‐SSS at the end of study decreased significantly from baseline (*p* < .001). IBS‐D‐SSS reduction was significantly different for the IgG diet + placebo and the IgG diet + probiotic (*p* < .001). (Table 3)

### 4.2 TIgG titer grade

Results for TIgG titers grade were consistent for both the Intention‐To‐Treat and Per‐Protocol analysis. At the end of study, TIgG titer grade was significantly decreased from baseline for each group (*p* < .001). However, there was no significant difference between IgG diet + placebo and IgG diet + probiotic, after intervention (Table 4).

### 4.3 Adverse events

Overall, the number of reported adverse events was small. Nine patients reported worsened gastrointestinal symptoms (two control, three Chi res diet, two IgG res diet, and two Chi + IgG res diet). Adverse events not related to diet were reported by 23% of the patients (e.g., headache, cold, and toothache), and were not different for diet (*p* = .316) or supplement groups (*p* = .388). No serious adverse events were reported.

## 5 DISCUSSION

All subjects enrolled in this study were from Harbin, the coldest provincial capital city in China. Local residents are accustomed to uncooked, cold, spicy, fried, and alcoholic foods in their daily diet. Many clinical IBS patients complain of abdominal symptoms induced by certain foods or certain properties of foods, which is similar to previous studies of patients in other areas.^12^ Patients automatically exclude some foods, but symptoms are often accidentally triggered by other foods. Therefore, it is necessary to explore the causes of IBS food intolerance to provide a more accurate method for food discrimination. This study is the first to explore a comparison of the effectiveness of a restricted diet from the perspective of food physical properties and IgG food positivity. A major discovery of this study was the identified interaction between the two diet restrictions. Both diet restrictions synergized to reduce IBS‐D‐SSS and decrease IgG antibody titer. For the IgG diet, there was no significant difference between the CSF diet and no CSF diet. Therefore, the IgG res diet had the greater effect. Although the two diet restrictions synergized, excessive dietary restriction can lead to intestinal flora imbalance and malnutrition. Hence, minimal diet restriction with appropriate and effective probiotics, allows for better patients compliance with diet restriction, minimizing flora imbalance and malnutrition.

Many studies have investigated a role for the intestinal microbiota in IBS.^22^ Probiotics have also been used clinically to improve the symptoms of IBS, yet the precise efficacy of probiotics for treatment of IBS is largely unknown. It is unclear whether particular combinations of probiotics are more effective than others, or whether there are particular IBS subtypes that are more responsive to probiotics.^26^ Interestingly, some effective probiotic preparations for the management of IBS symptoms contain Bifidobacteria,^24^ suggesting a potential role for Bifidobacteria in the treatment of IBS. Therefore, in the second part of this study, we recruited IBS‐D patients to complete a combined intervention of a food specific positive IgG restricted diet combined with a single strain of Bifidobacteria. Compared to the IgG diet+placebo, the IgG diet+Bifidobacteria probiotic significantly improved patient symptoms, even though TIgG titer was not significantly reduced.

In summary, these are the conclusions of this study. First, both the CSF res diet and the IgG res diet improved specific‐food IgG positive IBS‐D patient symptoms. Second, the IgG res diet was an important means by which to accurately eliminate certain foods. When intolerant foods cannot be eliminated, avoiding uncooked, cold, spicy, fried, and alcoholic foods is a good choice. Third, the effect of IgG food elimination combined with a probiotic is the best choice. Improvement in gastrointestinal symptoms by Bifidobacteria may be through a non‐IgG pathway.

Hidalgo‐Cantabrana et al.^27^ demonstrated *Bifidobacterium*‐mediated health benefits to be the result of a complex dynamic interplay among Bifidobacteria, other members of the gut microbiota, and the human host. This intricate interplay has not been fully deciphered at a molecular level, with current efforts seeking to understand the metabolic fluxes within the gut ecosystem that produce the microbiota‐host cross talk, which result in health or disease. Furthermore, it is worth noting that significant mutualistic effects have been described for Bifidobacteria and other intestinal bacteria.^28^ *In vitro* and in vivo studies have shown that modulating Bifidobacteria levels through probiotic or prebiotic supplementation can change the overall composition and metabolism of the gut microbiota.^29^ Further, the beneficial effect of Bifidobacteria on IBS symptoms may be due to the presence of serine protease inhibitors (SERPINs) in these bacteria.^30^, ^31^ Indeed, the mechanistic basis for the beneficial effect of Bifidobacteria on IBS patient symptoms requires further analysis.

There are limitations to this study. First, in the two parts of the study, 45% and 35% of patients were food IgG negative, respectively. Future research needs to expand specific IgG testing of more foods. Second, some studies have shown that the IgG4 subtype is closely related to IBS food intolerance and that specific subtype was not evaluated. It is worth nothing that the prevalence of positivity for IgG/IgA AGA in IBS patients has been reported to be 5%–17%^32^ or as high as about 50%.^33^ In the future we will assess additional markers to improve the accuracy of intolerant food elimination. Third, the effects of a variety of dietary restrictions for IBS patients may be due to different mechanisms of action. For example, a low‐FODMAPs diet reduces the symptoms and improves patient quality of life^34^, ^35^, ^36^, ^37^ by induction of favorable changes in the intestinal microbiota,^38^ fecal metabolome composition,^39^ and gastrointestinal endocrine cells.^40^, ^41^ More analysis of a variety of restricted diets and individualized patient treatments is necessary. Fourth, the detection of intestinal flora before and after dietary restriction can provide a reference for the treatment of probiotics and for the efficacy of probiotic treatment. Such detection is an essential component of future research. Fifth, probiotic combinations have been shown to improve overall IBS scores. Therefore, further research should focus on analysis of these combinations of probiotics at a fixed dose.

Restricted diets influence nutritional status, therefore the more accurate the determination of the food that requires restriction, the better the patient benefit and the more effective the patient's treatment. Further, understanding the means by which to use probiotics to improve bacterial imbalance caused by food restriction will also benefit patients. Therefore, in the future, we will guide patients through more accurate detection methods, precise restricted diets, and accurate probiotic intake, as our specific future direction.

## AUTHOR CONTRIBUTIONS

Xian‐Shu Zhao performed statistical analyses, acquired funding, reviewed and edited. Li‐Jun Shi designed and supervised. Bao‐Li Ning performed the execution and collected the data. Zhi‐Ming Zhao collected the data and wrote original draft. Xiao‐Xue Li performed experiments. Meng‐Hua Zhu performed experiments. Ya‐Bing Zhang performed experiments. Jun Fu designed the experiments, supervised, and acquired funding. All authors read and approved the final manuscript.

## CONFLICT OF INTEREST STATEMENT

The authors declare no conflict of interest.

## ETHICS STATEMENT

The ethics and protocol of this research study were assessed and approved by the Ethics committee of the First Affiliated Hospital of Harbin Medical University (No. 201324).

## Data Availability

The datasets used and/or analyzed during this study are available from the corresponding author on reasonable request.
